# Ship Biscuits

**Published:** 1875-02

**Authors:** 


### SHIP BISCUIT.

PILOT BREAD and crackers may be very advantageously kept on hand all the time in families, as they will keep fresh for months if made well and kept in a cool, dry place. If one of these, as large as a saucer and almost as hard as a piece of wood, is put in a plate, with boiling water poured on so as to just cover it, with another plate put over to keep in the steam, it becomes soft at once, and with butter spread over it, and a cup of tea, a good and healthful supper or breakfast is made for a dyspeptic; or, take a whole soda cracker in a cup plate, pour on boiling water enough to leave no residue, then put in the middle a teaspoonful of preserves or jelly or sauce, and a dessert is had, simple, healthful and nutritious, good enough for a king.

FIRE SCREENS.—It sometimes happens that an open fire makes it uncomfortably warm to those sitting near it, and yet it may not be convenient to sit farther away—as at a dinner table, for example. A glass screen on rollers would answer an excellent purpose, for while the cheerful light of the blazing fire passes through it, the heat of the fire is arrested; it cannot pass through the glass.

PRINCIPLES THREE.—The three great elementary principles of health in communities, in families and individuals are: perfect cleanliness, well-cooked food, and pure air. Reform in these respects must begin with the individnal. Each man must keep himself clean, his clothing, his apartment, his house and all its surroundings, up to his neighbor's line.

